# Mechanical cues enhance chondrocyte function: Insights from mechanoreception, regulation, and biological responses

**DOI:** 10.1016/j.mbm.2025.100164

**Published:** 2025-11-19

**Authors:** Gaige Wu, Shuai Chen, Qian Li, Min Zhang, Fuyang Cao, Junchao Wei, Li Guo, Pengcui Li, Xiaochun Wei, Quanyou Zhang

**Affiliations:** a Orthopaedic Laboratory, The Second Hospital of Shanxi Medical University, China; b College of Artificial Intelligence, Taiyuan University of Technology, China; c School of Basic Medical Sciences, Shanxi Medical University, China

**Keywords:** Biomechanics, Mechanoreception, Mechanoadaptation, Chondrocyte regeneration

## Abstract

Chondrocytes, the sole cell type in articular cartilage, are responsible for synthesizing and maintaining the primary components of the extracellular matrix (ECM). In daily life, chondrocytes are subjected to diverse mechanical stimuli, and the mechanoregulation of their biological responses plays a crucial role in cartilage function. Chondrocytes exhibit remarkable mechanoadaptation, as mechanical stimulation effectively promotes their homeostasis, development, and regeneration—critical factors for regenerative medicine. Thus, a deeper understanding of chondrocyte mechanosensing mechanism is essential. A key challenge lies in the significant biomechanical heterogeneity of chondrocytes across developmental stages and spatial locations of articular cartilage, leading to variations in their mechanosensing and mechanoresponsive behaviors. Elucidating the spatiotemporal biomechanical properties of chondrocytes is of great importance. Mechanical cues regulate chondrocyte homeostasis through multidimensional mechanisms, enhance energy metabolism, and dynamically couple with the cytoskeleton to optimize their responsiveness to matrix mechanical microenvironment. However, under pathological conditions, the aberrant mechanosensing of chondrocyte exacerbates inflammatory responses and matrix degradation, which further deteriorating the mechanical microenvironment. Growing evidence has indicated that some critical factors include dysregulated activation of mechanosensitive ion channels, disrupted integrin signaling pathways, and structural damage to primary cilia induce abnormal chondrocyte function. Biomechanical intervention strategies, such as mechanical loading techniques and exercise-based rehabilitation, hold promising potential for cartilage repair and regeneration by reconstructing the physiological-related mechanical microenvironment. This review provides a theoretical foundation for understanding the mechanisms of cartilage degenerative diseases and developing targeted therapies from a mechanobiological perspective.

## Graphical abstract

Abstract Figure: Overview diagram of mechanisms. Left box: Mechanical signals received by chondrocytes; Middle box: Mechanisms of mechanical signal reception and transmission; Right box: Physiological activities regulated by mechanical signals.

**Figure:**
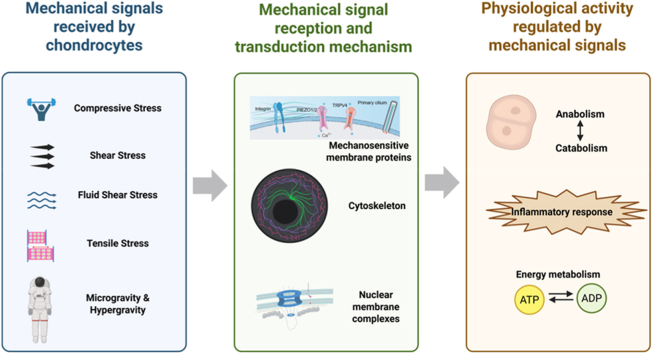

## 1 Introduction

In daily activities, articular cartilage is subjected to various mechanical stimuli, including compressive forces, tensile forces, shear stress, and fluid shear stress.^1^, ^2^, ^3^ Chondrocytes sense these mechanical signals through membrane-bound mechanoreceptors, converting mechanical cues into biochemical signals that activate pathways such as YAP/TAZ and MAPK, thereby regulating cell proliferation, differentiation, and extracellular matrix (ECM) synthesis.^4^ Mechanical forces maintain tissue homeostasis by modulating chondrocyte metabolic balance, with dynamic mechanical loading promoting cartilage morphogenesis during embryonic development and matrix renewal in the maturation stage. Additionally, appropriate cyclic stress activates the differentiation of chondrogenic precursor cells, and precise control of the mechanical microenvironment enhances chondrocyte regeneration.^5^, ^6^, ^7^, ^8^ Thus, understanding the mechanoregulative regulated biological responses of chondrocytes is critical for cartilage regeneration and repair.

The mechanosensing system of chondrocytes is cooperatively composed of membrane proteins, the cytoskeleton, and the nuclear membrane complex. Membrane proteins first sense extracellular mechanical forces, triggering calcium signaling or focal adhesion kinase (FAK) activation.^9^, ^10^, ^11^ The cytoskeleton dynamically reorganizes to transmit tension signals, regulating cell morphology and metabolic responses through mechanotransduction pathways such as the RhoA/ROCK pathway.^12^, ^13^, ^14^ The linker of nucleoskeleton and cytoskeleton (LINC) complex connects the cytoplasmic and nuclear skeletons, transmitting mechanical signals to the nucleus to activate YAP/TAZ transcription factors, thereby driving cartilage-specific gene expression.^15^^,^^16^ These three components form a “membrane-cytoskeleton-nucleus” mechanical signaling cascade. Through cross-scale integration of mechanical signals, they collectively regulate the mechanoadaptation of chondrocytes during development, homeostasis maintenance, and injury repair.

Under physiological conditions, dynamic mechanical loading activates relevant signaling pathways, driving chondrocyte proliferation and directional matrix deposition during development to promote cartilage morphogenesis. During cartilage maturation, such mechanical stimulus balances anabolic and catabolic processes to maintain articular cartilage health and function.^4^^,^^7^ Chondrocytes exhibit exceptional mechanoadaptation, with mechanical stimuli under physiological conditions promoting cellular homeostasis and optimizing energy metabolism.^17^, ^18^, ^19^, ^20^ Notably, chondrocytes display distinct biomechanical behaviors and biological responses to mechanical loads depending on their temporal developmental stages (e.g., embryonic, mature) and spatial positions (e.g., superficial, deep zones, chondron clusters) within articular cartilage.^21^, ^22^, ^23^ This spatiotemporal heterogeneity underscores the need for stage- and location-specific approaches in cartilage mechanobiological research and therapeutic interventions.

Moderate mechanical stress promotes cartilage metabolism, while abnormal mechanical stress triggers inflammation and finally leads to osteoarthritis (OA). In pathological conditions, the mechanoregulating biological response of chondrocytes manifests as a cascade of deteriorating mechanical microenvironments, aberrant mechanosensing, and disrupted homeostasis.^4^ Specifically, inflammatory factors (e.g., IL-1α) induce localized abnormal stress concentrations in chondrocytes, disrupting normal mechanical loading.^24^ Dysregulated mechanical signaling further causes imbalances in the RhoA/ROCK and YAP/TAZ mechanotransduction pathways,^25^^,^^26^ driving upregulated catabolism, inflammatory activation, and suppression of matrix synthesis, thereby forming a vicious cycle.^9^^,^^27^ The amplified mechanosensing dysfunction exacerbates homeostatic imbalance, ultimately accelerating cartilage degeneration and OA progression.

In this review, we examine the chondrocyte spatiotemporal biomechanical characteristics, their mechanosensors and the mechanoresponsive cell signalling under physiological and pathological conditions, with a particular emphasis on the temporal and spatial variations in chondrocyte mechanoadaptation. We emphasize how biomechanical regulation governs the biological responses of chondrocytes in health and disease, aiming to explore novel mechanical stimulation strategies to enhance chondrocyte regeneration and restore the mechanical properties of articular cartilage. Finally, we evaluate biomechanical stimulation approaches designed to promote chondrocyte regeneration, including dynamic compression, shear stress, and engineering matrix physical properties. These emerging techniques, grounded in spatiotemporally optimized mechanical cues, may hold therapeutic potential for clinical applications in cartilage repair and OA treatments.

## 2 Spatiotemporal mechanobiology of chondrocytes

### 2.1 Temporal variations in biomechanical characteristics of chondrocytes

The biomechanical properties of chondrocytes exhibit significant dynamic evolution throughout their lifespan, the main content can be divided into three stages (Table 1). Among them, juvenile chondrocytes demonstrate highly adaptable biomechanical behavior, characterized by nonlinear elasticity and exceptional viscoelasticity.^21^^,^^28^ The elastic modulus exhibits nonlinear attenuation with increasing indentation depth, stabilizing at a low value beyond a critical threshold. During minor deformations, chondrocytes display high stiffness, but their flexibility increases under large deformations to accommodate rapid developmental demands. Cell usually exhibits obvious viscoelastic behavior with the cytoplasm exhibiting high energy dissipation capacity, relying on a well-organized microfilament and microtubule network to maintain mechanical integrity. The cytoskeleton is densely packed and orderly arranged, enabling efficient mechanical signal transmission and supporting ECM synthesis.^29^^,^^30^ At this stage, cells respond sensitively to dynamic loading, activating cytoskeletal remodeling via the RhoA/ROCK pathway to promote matrix protein synthesis and tissue development.

**Table 1:** Temporal variations in biomechanical properties of chondrocytes.

Stage	Mechano-adaption	Mechano-sensing	Cellular mechanics	Structural organization	Mechanisms
Juvenile	Excellent	Sensitive	• Nonlinear elastic modulus • Significant viscoelastic characteristics	• High cytoskeletal density with well-organized microfilament and microtubule networks	The RhoA/ROCK pathway promotes cytoskeletal remodeling
Adult	Stabilize	Quality	• High elastic modulus • Superor viscoelastic properties	• Mature collagen-proglycan composite • Matrix and reinforced perinuclear ring structure of intermediate filaments	• The integrin-YAP/TAZ pathway integrates mechanical signals
Aging	Degradation	Poor	• Low elastic modulus • Loss of stiffness • Impaired viscoelasticity	• Atrophy of primary cilia and disorganization of the cytoskeleton	• The integrin-FAK • TGF-B/Smad • YAP/TAZ-Hippo pathway

In adult chondrocytes, mechanical properties stabilize, characterized by superior modulus retention capacity and metabolic-mechanical coupling.^21^^,^^29^ Maturation of the collagen-proteoglycan composite matrix and reinforcement of perinuclear ring-like structures formed by intermediate filaments (e.g., vimentin) enable the elastic modulus to remain elevated even under large deformations.^22^ Viscoelastic parameters are comparable to those in the juvenile stage, though energy dissipation efficiency slightly declines, reflecting steady-state adaptation after optimized matrix permeability and cytoskeletal fine-tuning.^29^ At this phase, cells efficiently integrate mechanical signals with mitochondrial oxidative phosphorylation via the integrin-YAP/TAZ pathway. Enhanced ATP synthesis efficiency under dynamic loading maintains the synthesis-degradation equilibrium of the ECM, supporting long-term joint function.^31^

In senescent or pathological process, the biomechanical properties of chondrocytes exhibit marked functional deterioration. The elastic modulus decreases under large deformations, accompanied by nuclear stiffness loss due to disintegration of perinuclear intermediate filaments, resulting in impaired compression resistance.^32^ Viscoelastic energy dissipation capacity is severely compromised, with diminished energy buffering capacity, aggravate the risk of mechanical damage.^29^^,^^30^ Cytoskeletal disorganization and atrophy of primary cilia compromise mechanosensing and signal transmission, which leads to aberrant activation of the PIEZO1-Ca^2+^-ROS pathway, and triggers overexpression of inflammatory factors and matrix degradation.^27^^,^^33^ Concomitantly, aberrant activation of transforming growth factor beta (TGF-β) can drive cellular fibrosis, inducing a further reduction in the viscoelasticity of cells and the ECM.^34^^,^^35^ On the ECM with deteriorated viscoelasticity, mechanical signals can be abnormally transduced through the integrin-cytoskeleton architecture, resulting in Ca^2+^ overload which regulates the nuclear translocation of YAP/TAZ.^34^ Concurrently, mitochondrial metabolic reprogramming further impairs the cellular response to mechanical stimuli, establishing a mechanics-metabolism vicious cycle. Ultimately, this cascade leads to cartilage degeneration.^36^^,^^37^ At this stage, the biomechanical characteristics of cartil are defined by structural instability, disrupted signal transduction, and reparative failure.

### 2.2 Spatial variations in biomechanical characteristics of chondrocytes

**Fig. 1:**
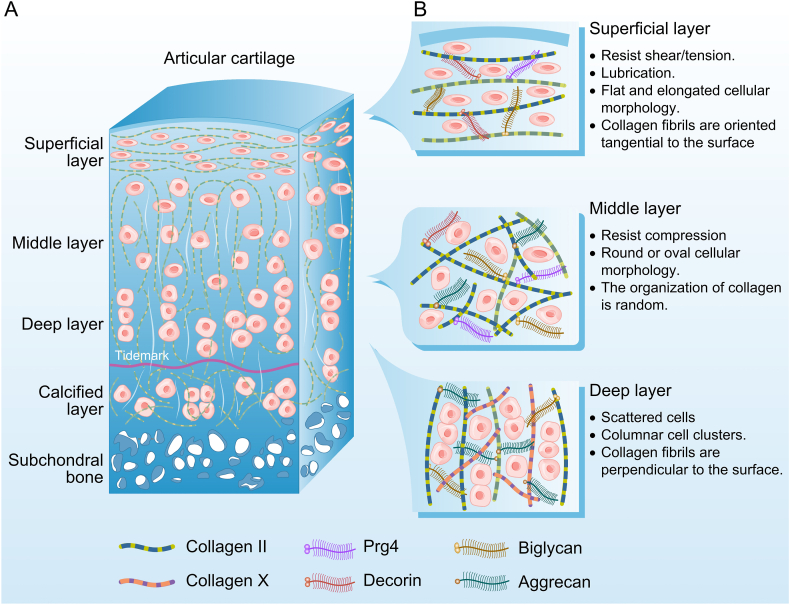
The layered structure and composition of the cartilage tissue of the knee joint. A. The layering of the cartilage of the knee joint. B. The arrangement of the cells and collagen in the articular cartilage layer.

Cartilage tissue exhibits unique spatially stratified mechanical properties: the superficial layer resists shear forces, the intermediate layer dissipates energy for cushioning, and the deep layer resists compression (Fig. 1). In the superficial layer, the chondrocytes are directly exposed to mechanical loads of the articular surfaces, exhibit biomechanical properties optimized for shear resistance and lubrication. These flattened spindle-shaped cells are embedded in a highly aligned collagen fiber network oriented parallel to the joint surface, forming a dense protective layer.^38^^,^^39^ In this region, cells withstand high shear stress and dynamic compressive loads, demonstrating pronounced deformational adaptability. At 10 ​% compressive strain, superficial chondrocytes show increased susceptibility to membrane blebbing and cytoplasmic vacuolization due to collagen fiber alignment, yet their F-actin stress fibers rapidly reorganize via the RhoA/ROCK pathway to stabilize membrane integrity.^40^^,^^41^ Excessive compression, however, disrupts cytoskeletal coherence, inducing aberrant PIEZO1 channel activation. This triggers pathological Ca^2+^ influx and inflammatory factor release, accelerating ECM degradation.^42^

Middle-layer chondrocytes, rounded or elliptical in shape, reside within proteoglycan-rich ECM, primarily responsible for compressive energy dissipation.^43^^,^^44^ The high osmotic pressure and low shear modulus of the ECM in this region confer superior viscoelasticity to the cells. Under static compression, perinuclear intermediate filaments (e.g., vimentin) form ring-like structures that evenly distribute compressive stress to the ECM, maintaining volumetric stability.^22^^,^^32^ During dynamic compression, the microtubule network facilitates cell volume recovery by regulating osmotic pressure, while fluid shear stress stimulates proteoglycan synthesis.^18^^,^^45^ Notably, middle-layer chondrocytes exhibit sensitivity to fluid shear stress, likely regulated through the Krüppel-like factor 4 (KLF4) and AMP-activated protein kinase (AMPK) pathways to modulate mitochondrial oxidative phosphorylation, thereby optimizing energy metabolism to support matrix synthesis.^46^^,^^47^

Deep-layer chondrocytes, aligned perpendicularly to the articular surface near the calcified cartilage interface, exhibit biomechanical properties dominated by compressive stress. These cells are embedded within vertically oriented collagen fiber bundles, where ECM stiffness is markedly elevated, forming a mechanical gradient to buffer stress at the bone-cartilage interface.^48^ Activating YAP/TAZ nuclear localization to drive expression of collagen cross-linking enzymes, thereby enhancing ECM compressive strength.^49^ Under pathological conditions (e.g., OA), abnormal calcification in deep-layer chondrocytes causes a drastic increase in ECM stiffness. This aberrant mechanical environment activates the PIEZO1-ROS-NF-κB pathway, promoting matrix metalloproteinases (MMP)-13 secretion and collagen network fragmentation.^9^^,^^27^^,^^50^

Shear stress from the superficial layer is transmitted to deeper regions via integrin β1-mediated mechanotransduction, triggering YAP-mediated matrix remodeling. Conversely, compressive loads in the deep layer regulate superficial-layer cell metabolism via fluid pressure modulation in the middle layer, thus forming a cross-layer mechanical signaling network. This spatial hierarchy of biomechanical properties, integrating structural specialization and dynamic interlayer communication, underpins the capacity of articular cartilage for efficient mechanical load-bearing and long-term functional integrity.

## 3 Mechanosensory components of chondrocytes

### 3.1 Mechanosensing mediated by membrane proteins

**Fig. 2:**
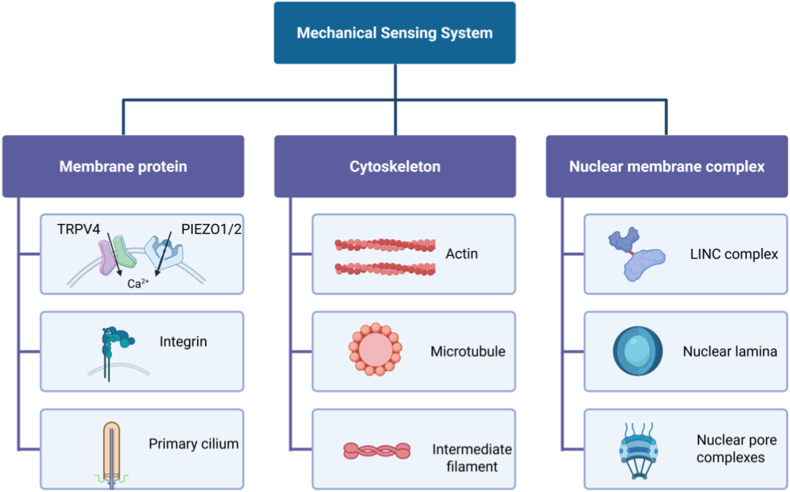
Chondrocyte mechanosensing components. Left: membrane protein (mechanosensitive ion channels: PIEZO1/2 andTRPV4, intergrin, primary cilia). Middle: Cytoskeleton (actin,microtubule and intermediate filament). Right: Nuclear membrane complex (LINC complex; Nuclear lamina; Nuclear pore complex).

Mechanical signals are integrated through a cascade pathway “membrane protein sensing - cytoskeletal transmission - nuclear membrane complex transduction (Fig. 2)”. Chondrocytes perceive external mechanical stimuli directly through membrane protein systems, with mechanosensitive ion channels serving as critical mechanosensors that regulate intracellular environments and signaling by controlling ion flux under mechanical forces.^9^^,^^10^ Transient Receptor potential (TRP) channels represent a major class of mechanosensitive ion channels. Notably, transient receptor potential vanilloid type 4 (TRPV4) responds to diverse mechanical cues such as stretching, compression, osmotic stress, and shear forces. TRPV4 activation mediates Ca^2+^ influx, maintaining intracellular Ca^2+^ levels and modulating chondrocyte anabolic activity under physiological mechanical stress.^51^^,^^52^ Similarly, PIEZO channels, a family of mechanosensitive cation channels, are Ca^2+^ permeable ion channels that are involved in chondrocyte mechanotransduction. PIEZO1 responds to compression, tension, and fluid shear stress, while PIEZO2 exhibits sustained activation under obesity-related weight-bearing pressure. In chondrocytes, both PIEZO1 and PIEZO2 not only act as direct mechanotransducers but also participate in inflammatory responses, cytoprotection, and pain sensation, linking mechanical signaling to diverse pathophysiological processes.^51^^,^^53^

Integrins are a class of heterodimeric transmembrane proteins composed of α and β subunits, acting as adhesion bridges between cells and the ECM. Their large extracellular domains mediate ECM ligand binding, while their smaller intracellular domains initiate signal transduction and anchor cytoskeletal proteins.^54^ Through the formation of focal adhesion (FA) complexes, integrins sense mechanical signals and coordinate the cellular response to the mechanical microenvironment, with the αV subtype playing a central role in this process.^11^ The αV integrin and its key adaptor protein Talin constitute a hub for the TGFβ1-mediated mechanical signaling pathway: On one hand, they directly sense extracellular mechanical stimuli triggered by changes in substrate stiffness^35^^,^^55^; On the other hand, Talin drives cytoskeletal reorganization, inducing cellular stiffening, FA remodeling, and cell polarization^56^^,^^57^; Concurrently, this complex activates downstream effector molecules, converting mechanical signals into metabolic reprogramming (such as enhanced glucose uptake and increased ATP synthesis) and functional responses.^35^^,^^58^ Notably, dysfunction of the integrin-talin axis can lead to aberrant mechanical signal transduction in articular cartilage, which is closely associated with the mechanical-load-dependent pathological activation of TGFβ in OA.^35^

Primary cilia, known as “cellular antennae” oriented toward the ECM, are microtubule-based structures (composed of a 9 ​+ ​0 microtubule arrangement, 3–5 ​μm in length) enveloped by the ciliary membrane, these organelles serve as mechanosensory hubs capable of detecting and transmitting mechanical signals.^59^, ^60^, ^61^ Mechanical stimuli alter primary ciliary morphology, inducing bending or length changes, which activate calcium channels (e.g., TRPV4 and PIEZO1) within the cilium. This results in elevated intracellular Ca^2+^ levels, thereby modulating cytoskeletal reorganization and matrix anabolism.^62^^,^^63^ Additionally, primary cilia sense fluid shear stress through bending, activating the Hedgehog signaling pathway to participate in cellular mechanosensation and mechanoregulation, and promote the proliferation and differentiation of chondrocytes.^64^^,^^65^

### 3.2 Dynamic mechanotransduction in the cytoskeleton

The cytoskeleton not only transmits mechanical signals through dynamic reorganization but also participates in mechanotransduction. When mechanical forces act on the cell membrane or cell-matrix adhesion sites (e.g., FA), actin stress fibers undergo remodeling, generating tension gradients.^12^ During this process, integrins detect mechanical signals by binding to the ECM, activating FA proteins to facilitate actin anchoring.^12^^,^^66^ Actin remodeling transmits mechanical signals to nuclear envelope-associated proteins. Concurrently, actin forms stress fibers that enhance tensile modulus, converting external forces into cellular contraction or spreading.^15^ Furthermore, mechanical stimulation activates ROCK kinase to amplify myosin II contractility, regulating actin dynamics to stabilize stress fibers and maintain cellular mechanoadaptation.^13^^,^^14^^,^^67^

Previously, the microtubule network was thought to lack direct responsiveness to mechanical stimuli compared to actin^68^, however, recent in vitro studies have demonstrated that mechanical forces influence microtubule structure, composition, and longevity. Researchers observed that application of compressive forces in living cells induced microtubule buckling, reduced dynamicity, and increased stability—a phenomenon termed “mechanostabilization”.^69^ This mechanostabilization enables microtubules to resist external forces, maintain structural integrity, and provide mechanical support during cellular dynamic processes such as cell migration and mitotic spindle orientation. Mechanical signals can propagate through the microtubule network to the nucleus or specific subcellular regions. Furthermore, microtubule mechanoresponses may synergize with the actin network via crosslinking proteins to activate Rho GTPases signaling pathways, amplifying mechanical signals and enabling feedback regulation.^68^^,^^70^ Additionally, mechanical stimuli may indirectly modulate cellular mechanoadaptation by altering post-translational modifications of microtubules, such as acetylation and tyrosination.^71^^,^^72^

Vimentin, a key member of the intermediate filament (IF) family and the predominant intermediate filament in chondrocytes, serves as a core regulator of cellular mechanical properties. It maintains homeostasis under physiological loading through structural support, mechanical signal transmission, and mechanotransduction.^73^^,^^74^ Studies reveal that healthy chondrocytes exhibit significantly higher global stiffness compared to OA chondrocytes. Disruption of the vimentin network markedly reduces stiffness in healthy cells but has minimal impact on OA chondrocytes, highlighting vimentin's critical role in preserving normal chondrocyte mechanical integrity.^32^ The vimentin network physically links the cell membrane to the nucleus, potentially acting as a “mechanical bridge” to transmit externally applied forces from the cell surface to the nuclear envelope, thereby influencing nuclear gene expression or epigenetic regulation.^73^^,^^75^ By maintaining cellular stiffness and regulating strain thresholds, vimentin modulates downstream signaling activation. Moreover, vimentin collaborates with the actin network to respond to dynamic compressive forces: actin mediates rapid mechanical strain responses, while vimentin provides long-term stability.^76^^,^^77^ Static compressive stimulation upregulates vimentin mRNA expression and promotes its filament assembly, suggesting that mechanical cues enhance cellular adaptation to sustained mechanical loading by modulating vimentin expression and network reorganization.^78^^,^^79^

**Fig. 3:**
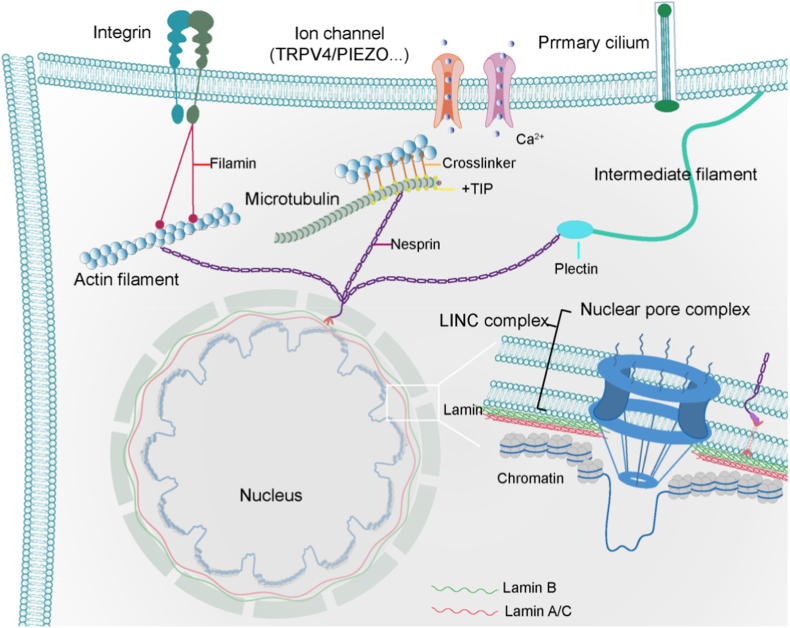
Chondrocyte mechanosensing regulatory protein interactions.

The three major cytoskeletal networks are highly coordinated rather than operating independently, the three cytoskeletal components constitute a hierarchical, interconnected, and dynamic system, as shown in Fig. 3. The actin network, leveraging its contractility and capacity for dynamic reorganization, leads to cellular movement and shape alteration. Actin bundles mediate directional microtubule growth through plus-end tracking proteins (+TIPs), while the cortical actin network stabilizes the microtubule network by anchoring both ends of microtubules and forms physical constraints that hinder microtubule penetration. The RHO GTPase signaling system coordinately regulates the dynamic changes of these dual networks.^68^^,^^80^^,^^81^ At the mechanical level, microtubules provide anti-retraction support within actin-driven membrane protrusions.^68^ IF forms deep mechanical integration with both actin and microtubules. Through specific linker proteins and physical intertwining with structures like the nuclear lamina (e.g., Lamin A/C), IF creates an integrated network. On one hand, their high extensibility allows them to absorb external stresses, protecting the fragile actin network. On the other hand, their rigid scaffold supports their own network distribution and buffers microtubules against mechanical shock.^69^^,^^82^^,^^83^ Plectin, acting as a core cross-network linker protein within hemidesmosomes, seamlessly connects all three cytoskeletal elements. This enables the complementary strengths–microtubule compression resistance, actin-generated driving force, and IF tensile strength–to reinforce each other.^82^^,^^84^^,^^85^ This multi-level cooperation allows the cell to precisely establish polarity, maintain mechanical stability, and resist deformation.

### 3.3 Mechanical-gene regulation of nuclear membrane complexes

The nuclear envelope complex, comprising the LINC complex, nuclear lamina, and nuclear pore complexes (Fig. 3), serves as a central hub for sensing, integrating mechanical signals, and regulating gene expression in the nucleus.^16^ The LINC complex connects the nuclear lamina at the inner nuclear membrane via SUN (Sad1 and UNC-84) -domain proteins and directly links to the cytoskeleton through KASH (Klarsicht, ANC-1, Syne homology) -domain proteins, forming a mechanical force-transmission pathway across the nuclear envelope.^86^ When external forces act on the cell, cytoskeletal-generated tension is transmitted through the LINC complex into the nucleus, inducing nuclear envelope deformation and triggering conformational changes in intranuclear mechanosensitive proteins. This activates downstream signaling pathways.^87^^,^^88^ For instance, under increased matrix stiffness, LINC-mediated tension propagates through the nuclear lamina to chromatin, promoting transcriptional activation of mechanosensitive genes (e.g., YAP/TAZ target genes), thereby regulating cell differentiation and migration.^89^^,^^90^ Furthermore, loss of the LINC complex causes nucleo-cytoplasmic mechanical uncoupling, impairing cellular responses to mechanical stimuli and leading to nuclear morphological abnormalities and gene misregulation.^91^

The nuclear lamina, primarily composed of lamin A and lamin B, determines the mechanical properties of the nucleus through its assembly state. The expression levels of lamins A/C determine the nuclear resistance to larger deformations, which induces nucleus strain stiffening.^92^ Under mechanical stress, it undergoes conformational changes that expose binding sites, recruiting chromatin remodeling complexes to perinuclear heterochromatin regions to repress or activate specific genes.^93^^,^^94^ For example, during fluid shear stress, increased tension in the nuclear lamina triggers phosphorylation of lamin A/C, releasing heterochromatin tethering and promoting transcription of mechanoresponsive genes such as COL1a1.^95^ In contrast, lamin B1 supports nuclear pore complex (NPC) anchoring by maintaining nuclear envelope integrity. Its loss leads to nuclear envelope crumpling and disrupted NPC distribution, impairing mechanical signal transmission to transcription factors.^96^^,^^97^ This mechanical force dynamic reorganization of the nuclear lamina directly couples external mechanical stimuli to epigenetic regulation.

NPC not only serve as nucleocytoplasmic transport channels but also act as mechanical sensors that respond to changes in nuclear membrane tension. Mechanically induced conformational changes in NPC can regulate the nuclear import efficiency of specific transcription factors, such as Smad and β-catenin. For instance, fluid shear stress enhances YAP nuclear import via NPC, activating pro-proliferative genes.^97^ Concurrently, NPC collaborates with the nuclear lamina to maintain tension homeostasis of the nuclear envelope. When the nuclear envelope is stretched, NPC regulates cytoskeletal reorganization by anchoring microtubule-organizing centers, thereby reciprocally influencing the cellular mechanical microenvironment.^98^^,^^99^ Additionally, mechanical stress-induced abnormalities in NPC components increase nuclear envelope permeability, leading to nuclear leakage of DNA damage repair factors and exacerbating mechanical stress-associated genomic instability.^97^ Through these multidimensional mechanical signal integration mechanisms, NPC emerge as critical hubs linking the cellular mechanical environment to intranuclear gene regulation.

Mechanical signals are integrated through a cascade pathway of “membrane protein sensing–cytoskeletal transmission–nuclear envelope complex transduction”. Membrane proteins sense mechanical cues and transmit them, the cytoskeleton propagates and amplifies these signals to the nuclear envelope complex, which ultimately drives mechanical environment-adapted gene expression. This coordinated mechanism ensures the mechanical adaptability of cartilage tissue. Under pathological conditions, dysregulation in any component of this pathway disrupts signaling, accelerating cartilage degeneration.

## 4 Mechanoregulation of chondrocyte biological responses

### 4.1 Mechanoregulation promotes chondrocyte homeostasis

**Fig. 4:**
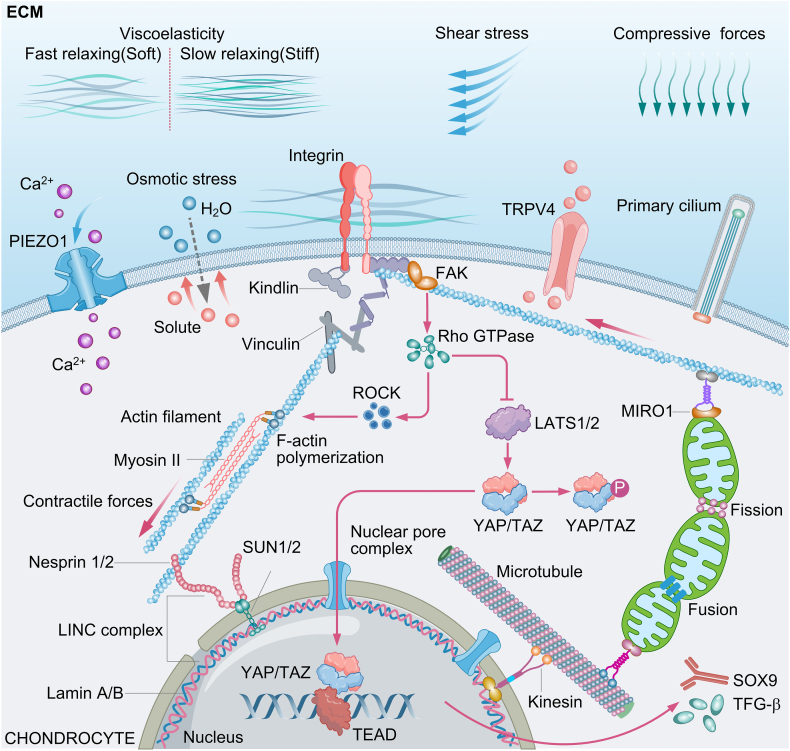
Chondrocyte homeostasis and energy metabolism homeostasis under mechanical regulation.

The biophysical microenvironment is essential for maintaining chondrocyte homeostasis, where physiological mechanical forces orchestrate ECM synthesis, cytoskeletal resistance to compressive forces, and mitochondrial oxidative metabolism through multidimensional molecular networks. This forms a mechano-bio-metabolic coupling system that safeguards tissue functionality (Fig. 4). Indeed, physiological mechanoregulation promotes chondrocyte homeostasis through multidimensional molecular networks. Under normal mechanical stimulation, chondrocytes perceive compressive and shear forces via mechanosensitive ion channels (e.g., TRPV4, PIEZO1) and integrin receptors, activating transcription factors such as SOX9 and YAP/TAZ, which drive the synthesis of type II collagen and proteoglycans.^11^^,^^51^ Dynamic compression and fluid shear stress enhance mitochondrial oxidative phosphorylation, providing an energetic foundation for matrix synthesis, while simultaneously facilitating proteoglycan assembly via integrin signaling pathways, thereby increasing proteoglycan content and optimizing the osmotic pressure regulation capacity of the ECM.^4^^,^^11^^,^^19^ Notably, the optimized osmotic pressure regulation in the cartilage ECM enhances fluid retention under intermittent shear stress, and elevated synthesis significantly improves the compressive modulus of cartilage.^43^^,^^47^^,^^100^ These biomechanical optimizations enable normal articular cartilage to maintain structural integrity even after millions of loading cycles, underscoring the critical role of mechanical microenvironments in regulating cartilage homeostasis.

Moderate mechanical signals can be sensed by integrins to activate the small GTPase RhoA, thereby activating its downstream kinase ROCK. ROCK phosphorylates and inhibits cofilin via LIM kinase (LIMK), promoting F-actin polymerization. Concurrently, ROCK enhances myosin II contractile activity. Together, these actions drive cellular stress fiber reorganization and tension gradient formation.^12^, ^13^, ^14^ This altered tension can downregulate the activity of the Hippo pathway kinases LATS1/2, suppressing the phosphorylation of YAP/TAZ. Simultaneously, changes in cellular tension facilitate the nuclear translocation of YAP/TAZ by enhancing the mechanotransduction efficiency of the nuclear pore complex. Subsequently, the dephosphorylated YAP/TAZ rapidly translocate into the nucleus and form a complex with the transcription factor TEAD.^15^ Within the nucleus, the YAP/TAZ-TEAD complex binds to TEAD-binding sites within the SOX9 gene promoter region, activating SOX9 transcription and upregulating SOX9 expression.^101^ Furthermore, this complex can induce TGF-β synthesis and upregulate the expression of anabolic genes.^102^

In the dynamic remodeling of the cytoskeleton, F-actin forms stress fibers via the RhoA/ROCK pathway, while intermediate filaments assemble into a compression-resistant ring around the nucleus, conferring mechanical stability to the cell. This coordinated alignment of collagen fibers synergistically enhances the fatigue resistance of chondrocytes.^14^^,^^32^ Such mechanobiology coupling mechanisms enable cartilage to dissipate energy through viscoelastic strain under cyclic loading, maintaining a balance between matrix synthesis and catabolism. This homeostatic state relies on precise interactions among mechanical signals, metabolic pathways, biomechanical microenvironments, and cytoskeletal reorganization.

### 4.2 Mechanoregulation for optimize energy metabolism

Under normal mechanical stimulation, chondrocytes activate the AMPK/Sirtuins-PGC-1α pathway via integrin receptors and mechanosensitive ion channels (e.g., TRPV4, PIEZO1), significantly enhancing mitochondrial oxidative phosphorylation capacity.^103^^,^^104^ Dynamic compression or fluid shear stress promotes Ca^2+^ influx, activating pyruvate dehydrogenase to accelerate the conversion of pyruvate to acetyl-CoA, thereby driving tricarboxylic acid (TCA) cycle activity while suppressing HIF-1α-mediated glycolytic metabolism.^105^^,^^106^ This metabolic shift enhances mitochondrial respiratory chain complex activity, improves ATP synthesis efficiency, and the process is accompanied by elevated mitochondrial membrane potential and a moderate increase in ROS signaling, which activates antioxidant enzymes to maintain redox homeostasis.^19^^,^^107^ Furthermore, mechanical stimulation promotes mitochondrial biogenesis through the YAP/TAZ pathway, forming a highly plastic mitochondrial network that optimizes energy supply to meet dynamic loading demands.^19^^,^^31^

Mechanical stimulation activates YAP/TAZ through integrin-cytoskeletal signaling, directly upregulating PGC-1α expression to enhance mitochondrial oxidative metabolism. Concurrently, ATP generated by mitochondria feedback-regulates PIEZO1 channel activity via AMPK phosphorylation cascades, forming a closed-loop regulatory system that integrates metabolic and mechanical signaling.^46^^,^^108^^,^^109^ Under moderate loading, mitochondrial ROS acts as a secondary messenger to activate protective autophagy, clearing damaged mitochondria and promoting metabolic adaptation. This interplay maintains a dynamic balance between energy metabolism and mechanical responsiveness. However, abnormal mechanical loading disrupts this equilibrium, inducing excessive mitochondrial fission, electron transport chain uncoupling, and compensatory upregulation of glycolysis. These alterations trigger NAD+/NADH imbalance and depletion of metabolic reserves, ultimately exacerbating mitochondrial dysfunction through a Ca^2+^-ROS positive feedback loop.^37^

## 5 Mechanoregulation and biological responses of chondrocytes in the pathological process

### 5.1 Mechanical microenvironment degeneration of chondrocytes

**Fig. 5:**
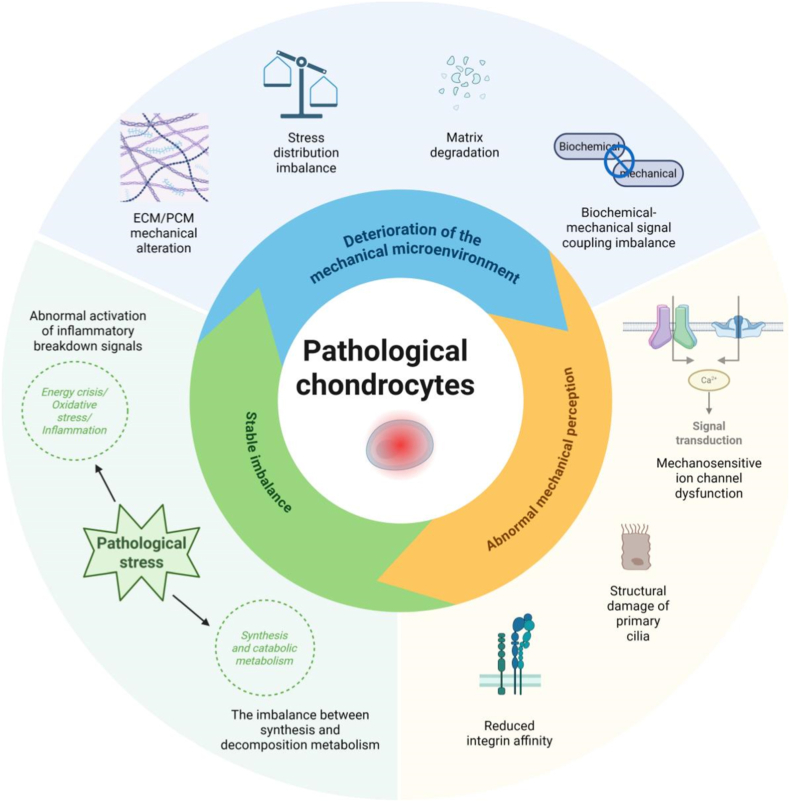
The mechanical regulation and biological responses of chondrocytes in the pathological process.

The pathological biomechanical microenvironment in osteoarthritis disrupts cellular mechanosensing, triggering calcium overload that drives mitochondrial damage, metabolic collapse, oxidative stress, and an inflammatory cytokine storm. This cascade further suppresses SOX9 synthesis, promotes catabolism, and induces cellular senescence, establishing a self-perpetuating vicious cycle that ultimately leads to complete collapse of cartilage homeostasis (Fig. 5). Under pathological conditions, the chondrocyte mechanical microenvironment undergoes a multi-layered dynamic deterioration, involving altered biomechanical properties of the ECM and pericellular matrix (PCM), imbalanced stress distribution, and dysregulated coupling of mechanochemical signaling. In early OA, the stiffness of the cartilage PCM rapidly declines, significantly compromising its mechanical support function. As the PCM serves as a mechanical buffer between cells and the ECM, its reduced elasticity directly alters local stress distribution around cells, inducing abnormal stress concentration.^110^ Concurrently, the ECM in early OA exhibits abnormally increased stiffness due to proteoglycan loss and collagen disorganization. However, this stiffness elevation lacks heterogeneity, exacerbating localized stress disparities across joint surfaces and promoting microcrack formation.^50^ As the disease progresses to advanced stages, both the ECM and PCM experience a marked reduction in stiffness, leading to loss of compressive and shear resistance in cartilage tissue, thereby exposing cells to supraphysiological mechanical loads.^111^ This biomechanical degeneration is closely linked to collagen network disruption, hyaluronic acid degradation, and disrupted hydration, forming a self-reinforcing vicious cycle.

The deterioration of the mechanical microenvironment is further manifested by abnormal fluid shear stress and synergistic damage from matrix degradation products. The decline in joint lubrication function alters the viscosity of synovial fluid, exposing cartilage surfaces to fluid shear stress intensities beyond the normal physiological range.^112^ Aberrant shear stress triggers sustained Ca^2+^ influx via PIEZO1 channel activation, leading to mitochondrial membrane potential depolarization, ROS bursts, and disruption of cytoskeletal stability, which exacerbates membrane ruffling and intracellular transport dysfunction.^113^^,^^114^ Concurrently, damage-associated molecules such as fibronectin fragments and hyaluronic acid oligomers—products of ECM degradation—penetrate the weakened PCM barrier, binding to cell-surface TLR2/4 receptors to activate inflammatory pathways like NF-κB.^115^^,^^116^ This synergy between mechanical overload and inflammatory mediators creates a vicious cycle: Abnormal mechanical signaling upregulates MMP-13 and a disintegrin and metalloproteinase with thrombospondin motifs (ADAMTS)-5 expression to accelerate matrix catabolism, while inflammatory cytokines (e.g., IL-1β, TNF-α) suppress integrin signaling, impairing cellular adaptive remodeling in response to mechanical stimuli.^117^ Ultimately, the systemic collapse of the mechanical microenvironment deprives chondrocytes of the physical foundation for homeostatic regulation, driving irreversible degenerative progression.

### 5.2 Abnormal mechanosensing of chondrocytes

Under pathological conditions, the mechanosensory system of chondrocytes becomes dysfunctional, primarily characterized by dysregulation of mechanosensitive ion channels, structural damage to primary cilia, and aberrant integrin signaling transduction. First, the dysregulation of mechanosensitive ion channels significantly impairs mechanochemical signal conversion. In OA, TRPV4 channels exhibit abnormal upregulation and reduced activation thresholds, driving sustained Ca^2+^ influx that induces mitochondrial membrane potential depolarization, ROS bursts, and upregulation of inflammatory factors, while simultaneously suppressing SOX9-mediated type II collagen synthesis.^27^^,^^118^, ^119^, ^120^ Conversely, PIEZO1 undergoes excessive activation under aberrant shear stress, suppressing autophagic clearance via mTORC1 signaling to promote the accumulation of senescence markers (e.g., P16, SA-β-gal). Additionally, PIEZO1 hyperactivation drives fibrotic phenotypes through YAP/TAZ nuclear translocation, directly disrupting cartilage-specific ECM organization^33^^,^^121^, ^122^, ^123^。

Furthermore, structural damage to primary cilia and dysregulation of integrin signaling pathways exacerbate mechanosensory deficits. During OA progression, primary cilia exhibit reduced numbers, shortened lengths, and disorganized localization, impairing the effective activation of the Hedgehog signaling pathway in response to mechanical stimuli. This renders chondrocytes incapable of directional responses to compressive forces.^60^^,^^64^^,^^65^ Concurrently, decreased expression of the integrin α5β1 subunit reduces its binding affinity to ECM ligands, thereby diminishing the activation efficiency of the FAK pathway. This failure to convert mechanical stimuli into anabolic signals disrupts ECM maintenance.^124^^,^^125^ These mechanosensing abnormalities form a positive feedback loop with cytoskeletal remodeling, further compromising the spatial coordination of mechanical signaling. Ultimately, chondrocytes lose their capacity to regulate mechanical homeostasis, accelerating degenerative progression.^25^^,^^32^^,^^114^^,^^126^

### 5.3 Pathological stress-induced disturbance of chondrocyte homeostasis

Under pathological conditions, chondrocytes exhibit a metabolically disordered state marked by aberrant activation of inflammatory catabolic signaling. Firstly, energy metabolic reprogramming and mitochondrial dysfunction are central features of homeostatic imbalance. Abnormal mechanical stimuli trigger sustained Ca^2+^ influx through TRPV4/PIEZO1 channels, leading to decreased mitochondrial membrane potential and reduced oxidative phosphorylation efficiency, forcing cells to switch to glycolysis for energy production.^127^^,^^128^ However, increased glycolytic flux is accompanied by excessive lactate accumulation, further acidifying the microenvironment and activating the HIF-1α pathway, which suppresses SOX9-mediated synthesis of type II collagen and aggrecan.^129^ Concurrently, mitochondrial ROS bursts not only directly damage mitochondrial DNA and protein function but also drive inflammatory cytokine release via NLRP3 inflammasome activation, creating a cascading interplay of “energy crisis-oxidative stress-inflammation”.^52^^,^^130^

Secondly, the imbalance between anabolic and catabolic processes, along with altered cell fate decisions, exacerbates tissue destruction. Pathological mechanical stimuli prolong Ca^2+^ influx via PIEZO1, activating the mTORC1 pathway to suppress autophagy while promoting cellular senescence and apoptosis.^33^^,^^121^ Concurrently, YAP/TAZ nuclear translocation drives fibrotic phenotypes, inducing aberrant deposition of type I collagen and suppressing type II collagen synthesis, thereby disrupting the mechanical integrity of cartilage ECM.^101^^,^^123^^,^^131^ In catabolic processes, abnormal mechanical signals synergize with inflammatory cytokines to activate NF-κB and MAPK pathways, upregulating MMP-13 and ADAMTS-5 expression to accelerate collagen network fragmentation and proteoglycan loss.^120^^,^^132^, ^133^, ^134^ Additionally, the senescence-associated secretory phenotype (SASP) recruits immune cell infiltration, amplifying the inflammatory response and suppressing the reparative capacity of neighboring chondrocytes.^135^^,^^136^

Ultimately, this multidimensional homeostatic imbalance creates a self-reinforcing vicious cycle. Metabolic abnormalities compromise cellular repair capacity, catabolic processes exacerbate ECM degradation, and the inflammatory microenvironment further distorts mechanosignal transduction, rendering chondrocytes incapable of adapting to physiological loads. The persistent disruption of mechano-biochemical coupling drives cartilage tissue from localized degeneration to full-thickness defects, culminating in irreversible joint dysfunction.

## 6 An attempt to biomechanically promote chondrocyte regeneration

**Fig. 6:**
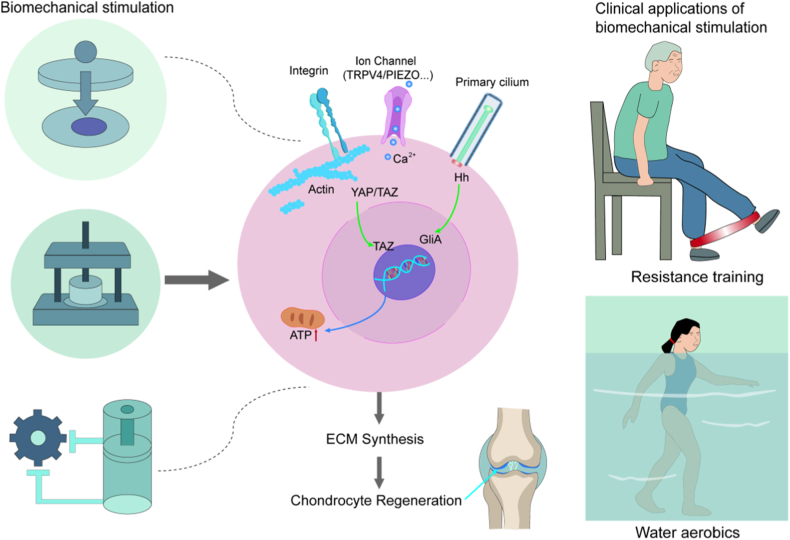
Basic applications and clinical translation of chondrocyte repair and regeneration based on biomechanics.

Biomechanical loading techniques significantly enhance cellular function and tissue regeneration in cartilage repair and regeneration by mimicking or modulating the mechanical microenvironment of cartilage tissue (Fig. 6). Dynamic mechanical loading activates TRPV4/PIEZO1 ion channels to trigger Ca^2+^ influx, synergizing with the integrin-cytoskeleton network to enhance YAP/TAZ nuclear localization and promote SOX9 and TGF-β/Smad pathway activation.^9^^,^^15^^,^^51^ This mechano-biochemical coupling improves the synthesis efficiency of type II collagen and proteoglycans. Intermittent compression at specific frequencies regulates primary cilium length, amplifying Hedgehog signaling transduction and promoting chondrocyte proliferation and differentiation.^137^^,^^138^

During regeneration, mechanical stimulation dynamically regulates energy metabolism. Moderate dynamic compression activates mitochondrial oxidative phosphorylation, increasing ATP synthesis rates while inhibiting key glycolytic enzymes, thereby providing sufficient energy for ECM synthesis.^4^^,^^108^ Notably, mechanical stimuli upregulate HIF-1α-mediated VEGF inhibitory signaling, effectively blocking pathological vascular invasion and creating an immune-privileged microenvironment conducive to cartilage regeneration.^139^, ^140^, ^141^ Advanced biomechanical intervention strategies, such as exercise-induced piezoelectric stimulation, activate PIEZO1 and TGF-β1 pathways to enhance cell migration and promote cartilage repair.^142^^,^^143^ Furthermore, when bioreactor systems are combined with three-dimensional scaffolds for chondrocyte culture, mechanical stimulation enables engineered cartilage to achieve compressive moduli comparable to native tissue. Surface motion promotes superficial zone protein and hyaluronic acid production, while compressive loading enhances type II collagen synthesis.^144^, ^145^, ^146^

## 7 Clinical attempts to promote chondrocyte regeneration by biomechanical strategies

Under pathological conditions, abnormal joint alignment can lead to deterioration of the cartilage's mechanical microenvironment. This triggers chondrocytes to respond abnormally to mechanical signals, resulting in reduced anabolic processes and increased catabolic processes and inflammatory factor release. Ultimately, this leads to the progressive destruction of articular cartilage. In clinical applications, biomechanical stimulation protocols primarily achieve cartilage protection and regeneration through customized exercise rehabilitation. The core mechanism lies in restoring chondrocyte metabolic balance, suppressing inflammatory damage, and alleviating joint pain through mechanical correction, appropriate mechanical load regulation, and selection of suitable mechanical patterns. For different degenerative joint diseases, clinicians employ differentiated exercise regimens. For patients with anterior cruciate ligament (ACL) injuries, early-intervention exercise rehabilitation can improve knee joint stability. Observation of clinical outcome indicators over two years confirms that exercise therapy can improve the prognosis of patients with ACL injuries by alleviating knee symptoms.^147^^,^^148^ In degenerative meniscal tear patients, low-intensity aerobic exercise combined with biomechanical gait correction training delays joint space narrowing progression, achieving comparable effects to arthroscopic surgery while avoiding operative risks.^149^ For patients with knee OA experiencing impairment of the cartilage tissue's mechanical microenvironment, moderate exercise with reduced joint loading leads to a significant increase in the protective anti-inflammatory cytokine IL-10 within the joint synovial fluid (p ​< ​0.05),^150^^,^^151^ while significantly decrease the level of cartilage oligomeric matrix protein (COMP) in the synovial fluid post-exercise (p ​< ​0.05).^150^ In another 12-week clinical trial study, non-weight-bearing quadriceps strengthening exercises reduced pain scores from 5.9 ​± ​1.4 to 4.1 ​± ​2.6, and weight-bearing functional exercises reduced pain scores from 5.9 ​± ​1.2 to 3.4 ± 1.9.^152^

It is noteworthy that biomechanical load control is critical, and current clinical practice emphasizes biomechanical appropriateness. In postmenopausal women, moderate-intensity walking combined with balance training demonstrated a 7 ​% difference in T2 relaxation time changes of full-thickness patellar cartilage after 12 months, indicating a favorably modulated state of cartilage matrix hydration following exercise intervention.^153^ For obese patients, aquatic exercise is employed to reduce joint loading, where buoyancy offsets partial body weight, thereby decreasing cartilage compression while maintaining muscle strength. Wearable real-time joint torque monitoring enables dynamic adjustment of exercise intensity to prevent abnormal stress concentration.^154^, ^155^, ^156^ Tailored to patient age and disease stage, early-stage OA management prioritizes low-intensity cyclic stimulation, whereas advanced stages focus on preserving joint mobility and compensatory periarticular muscle training.^157^^,^^158^ These strategies significantly elevate cartilage matrix metabolic biomarker levels and effectively delay disease progression.

In summary, clinical biomechanical interventions promote cartilage repair and regeneration through mechanobiology-coupled mechanisms. Their efficacy relies on personalized loading protocols and precise implementation. Future advancements should integrate intelligent monitoring technologies to further optimize therapeutic regimens. Inertial sensors have now been successfully utilized to assess the presence and severity of OA, evaluate the risk of disease progression, and provide feedback for gait retraining.^159^ In a 6-week clinical study, sensor-based gait retraining effectively reduced medial knee load, alleviated knee pain, and improved symptoms in patients with early-stage medial compartment knee OA.^160^

## 8 Conclusions and perspectives

This review systematically elucidates the mechanosensing mechanisms of chondrocytes under mechanical loading, their biomechanically regulated biological responses, and dynamic evolution patterns under physiological and pathological conditions. Chondrocytes transduce mechanical signals through cross-scale mechanotransduction systems spanning “membrane proteins-cytoskeleton-nuclear envelope”. We emphasize the temporal developmental and spatially zoned biomechanical heterogeneity of articular chondrocytes, particularly focusing on their mechanical behavioral characteristics. Postnatal juvenile chondrocytes exhibit biomechanically adaptive features dominated by nonlinear elasticity and viscoelasticity, while mature chondrocytes stabilize their mechanical properties, maintaining superior elastic modulus and excelling in mechanobiological metabolic conversion. The spatially graded biomechanical properties of mature chondrocytes reflect their specialized adaptations to distinct biomechanical microenvironments. However, chondrocyte mechanoadaptation has inherent limitations—damage to any component of the mechanotransduction cascade can disrupt signaling and accelerate cartilage degeneration. Current understanding of chondrocyte mechanical microenvironments and force transmission behaviors remains incomplete, necessitating novel methodologies to address these biomechanical challenges.

Chondrocytes still face multiple challenges in biomechanical adaptation. For instance, the mode-specific activation thresholds of TRPV4 and PIEZ01 channels and their dynamic interaction with LINC complexes in decoding mechanical signals remain unclear. The molecular mechanisms by which degraded ECM products in pathological microenvironments disrupt integrin-FAK signaling to induce abnormal cytoplasmic retention of YAP/TAZ have not been fully elucidated. Personalized mechanical interventions lack precise calibration while real-time stress monitoring technologies remain inadequate. Moreover, the crosstalk nodes between mechanical stimulation-driven anabolism and catabolism require optimization of mechanical parameters to achieve dynamic equilibrium, necessitating innovative breakthroughs integrating single-cell mechanomics and cell-instructive biomaterial development.

The influence of inflammatory microenvironments on chondrocyte biomechanical adaptation must also be considered. Inflammatory conditions significantly distort chondrocyte mechanoresponses: IL-1β/TNF-α inhibits integrin α5β1-FAK phosphorylation to block mechanotransduction while activating the NF-κB pathway to upregulate MMP-13, accelerating ECM degradation. These cytokines further induce mitochondrial membrane potential collapse via ROS overproduction, leading to abnormal nuclear retention of YAP/TAZ and suppression of SOX9-mediated type II collagen synthesis, establishing a vicious cycle of “mechanodesensitization-enhanced catabolism”. Additionally, ECM degradation products like hyaluronan oligomers can bind TLR4, synergizing with abnormal shear stress to perpetuate inflammasome activation. This necessitates the development of dual-intervention strategies combining mechanical loading with anti-inflammatory targeting to restore mechano-biological coupling equilibrium.

Future research should focus on the pivotal role of precise biomechanical modulation in cartilage regeneration. By deciphering multi-scale mechanotransduction mechanisms, developing mechanoresponsive smart biomaterials, 3D bioprinting and technologies Joint-on-chip, researchers can construct biomimetic mechanical microenvironments to directionally induce cartilage matrix synthesis. Concurrently, integrating machine learning with personalized gait biomechanics and joint inflammatory profiles will enable the establishment of dynamic loading algorithms to disrupt the vicious cycle of pathological mechanics and inflammation. Ultimately, this will achieve a multi-level synergistic therapeutic strategy encompassing “mechanical microenvironment remodeling - cell fate programming - functional ECM regeneration”, offering a novel regenerative medicine paradigm that combines biological compatibility with clinical feasibility for repairing degenerative cartilage.

## CRediT authorship contribution statement

**Gaige Wu:** Writing – review & editing, Writing – original draft, Conceptualization. **Shuai Chen:** Visualization, Conceptualization. **Qian Li:** Visualization. **Min Zhang:** Writing – review & editing, Visualization, Resources, Conceptualization. **Fuyang Cao:** Writing – review & editing, Methodology, Investigation, Data curation. **Junchao Wei:** Writing – review & editing, Supervision, Formal analysis, Conceptualization. **Li Guo:** Writing – review & editing, Supervision. **Pengcui Li:** Visualization, Supervision, Resources, Data curation, Conceptualization. **Xiaochun Wei:** Writing – review & editing, Supervision, Resources, Project administration, Conceptualization. **Quanyou Zhang:** Writing – review & editing, Visualization, Resources, Project administration.

## Ethical approval

This study does not contain any studies with human or animal subjects performed by any of the authors.

## Declaration of competing interest

The authors declare that they have no known competing financial interests or personal relationships that could have appeared to influence the work reported in this paper.
